# Training the Digital Clinician by Evaluating Health Education and Curriculum Integration New Zealand Psychology and Psychiatry Programs: Mixed Methods Study

**DOI:** 10.2196/72777

**Published:** 2025-12-31

**Authors:** Catherine Rawnsley, Karolina Stasiak

**Affiliations:** 1 Department of Psychological Medicine, University of Auckland, 28 Park Ave, Grafton, Auckland, 1023, New Zealand, 64 93737599 ext 83890

**Keywords:** digital tools, eHealth, mental health, mixed methods, training, workforce

## Abstract

**Background:**

The importance of digital health education is widely recognized; however, structural and knowledge deficits hinder its effective integration into training and on-the-job upskilling programs. Tackling these challenges will equip clinicians to navigate the fast-evolving digital mental health landscape confidently.

**Objective:**

This study aims to investigate the prevalence of digital health education and training needs for New Zealand mental health clinicians and trainees, including how psychology and psychiatry teaching programs are including eHealth and digital mental health tools in their curriculums.

**Methods:**

A mixed method study was conducted between August 2021 and February 2022: (1) a survey of mental health clinicians and trainees investigating existing and desired training in digital mental health tools, (2) follow-up in-depth one-on-one interviews with a subsample of survey participants, and (3) in-depth one-on-one interviews with educators (program or curriculum coordinators) within psychology and psychiatry training programs.

**Results:**

The study comprised a survey of 118 clinicians, follow-up interviews with 17 clinicians, and interviews with 4 program directors of relevant training programs. The survey results revealed that 75% (n=88) of the clinicians had not received formal digital health training, yet 69% (n=81) had engaged in self-directed learning. Interest in further training was strong, with 83% (n=98) expressing moderate-to-high interest. Two key themes emerged from the clinician interviews: (1) *openness to upskilling*, reflecting a willingness to learn, and (2) *barriers of time and leadership*, highlighting challenges in accessing training due to workloads and limited institutional support. From the program director interviews, three themes were identified: (1) *curriculum overload*, reflecting difficulties incorporating new content into already crowded programs; (2) *uncertainty and inconsistency*, with educators unsure about the scope and delivery of digital mental health education; and (3) *growth and future potential*, highlighting optimism about integrating digital health training into curricula.

**Conclusions:**

The findings reveal a pressing gap in formal digital health training for clinicians despite widespread interest and enthusiasm for upskilling. Key barriers—time constraints, limited institutional leadership, and a lack of educator expertise—are slowing progress.

## Introduction

Training is a fundamental component of clinicians’ education and professional development, ensuring they acquire the necessary skills to provide high-quality care. Formal education includes theoretical instruction, practical skill development, and supervised placements that build competencies in clinical decision-making, patient communication, and ethical practice [1]. However, digital health training remains inconsistently integrated into professional degree programs, leaving many graduates underprepared for the increasing role of technology in health care. Beyond their initial qualification, clinicians engage in ongoing professional development, including on-the-job training, continuing education programs, and peer learning, to stay updated on new treatments, technologies, and best practice guidelines [1].

A growing body of research highlights that a lack of training and familiarity with digital health is a key barrier to adoption for clinicians and clients [2-4]. Studies investigating perceptions of digital tools—whether through surveys of university students [5], clinicians [6], evaluations of new digital interventions [7], or the implementation of digital clinics [8]—consistently emphasize the importance of training in shaping confidence and engagement. Without structured education in digital health, clinicians may struggle to assess, implement, or recommend these interventions effectively, limiting their integration into routine practice.

Given the breadth of digital health, training must encompass a range of competencies, including telehealth, patient portals, electronic health records, mobile apps, and ethical considerations related to digital care delivery. In response, professional organizations and regulatory bodies across jurisdictions, such as the United States, Canada, and the United Kingdom, have begun developing guidelines, competency frameworks, and position statements on digital health [9-11]. However, these vary widely in scope and implementation, creating inconsistencies in training standards. In New Zealand, where this research is based, the Ministry of Health has developed a national digital health strategy to enhance health care accessibility and quality, accompanied by government-funded initiatives supporting the development and evaluation of digital interventions [12].

Despite these policy endorsements and technological advancements, integrating digital mental health tools into clinical practice has been slower than expected [13]. While components such as electronic health records have been widely adopted, digital mental health interventions remain underutilized, representing an unrealized opportunity to improve access, efficiency, and outcomes. A critical barrier is the lack of structured eHealth education. Evidence suggests that targeted training can address these gaps, fostering greater acceptance and use of digital health tools [2,14]. However, clinicians must also adapt their workflows, embrace emerging technologies, and develop the competencies required for effective engagement [15,16].

Existing literature reveals a significant gap in digital health education, with few university programs or clinical training pathways explicitly incorporating eHealth competencies [5,11,17]. Where digital health has been introduced into training, studies consistently show improved knowledge, increased confidence, and more positive attitudes among students and clinicians [5]. However, several challenges remain in delivering digital health education, including the rapid pace of technological change, inconsistent terminology, a lack of standardized guidelines, and the vast scope of the field [10]. Further research is needed to determine clinicians’ specific training needs, identify gaps in existing curricula, and explore the barriers preventing the widespread adoption of digital health.

Decisions regarding whether digital health training should occur during formal education or as part of continuing professional development are critical. It remains unclear whether academic institutions, health care organizations, or independent providers are best placed to deliver this training. Addressing these issues is essential to ensure clinicians are adequately equipped to integrate digital interventions into practice. Structured training and capacity-building initiatives will be key to overcoming clinician hesitancy, strengthening workforce readiness, and embedding digital health into routine mental health care [3,18-20].

This paper is part of a broader research program examining the integration of digital mental health tools into clinical practice in New Zealand. While previous studies [21,22] have explored uptake, attitudes, and user experiences with digital interventions, this paper focuses specifically on the training needs and availability of eHealth education for clinicians and trainees. This study aims to identify gaps, barriers, and opportunities to enhance training provision by investigating how psychology and psychiatry training programs incorporate digital health components. The findings will inform future policy and practice, supporting the effective integration of digital tools into mental health care.

## Methods

### Ethical Considerations

Ethical approval was obtained from the University of Auckland Human Participants Ethics Committee (reference: UAHPEC22599). Participants in Part One were offered to go into a draw for one of three $50 New Zealand dollar gift vouchers (approximately US $30), and those who were interviewed in Part Two or Part Three were offered a $20 New Zealand dollar gift voucher (approximately US $10) for participating. Participant information sheets were available prior to data collection to ensure informed consent, and any identifiable information was removed during data analysis to ensure anonymity.

### Participants and Procedure

A mixed method study was conducted between August 2021 and January 2022 to examine the training and education needs for digital mental health tools in New Zealand. The study consisted of three parts: (1) an online questionnaire, (2) follow-up interviews offered to those who had completed the online survey, and (3) semistructured interviews with educators from psychology and psychiatry training programs. This paper presents findings relevant to education and curriculum needs identified across the 3 studies. Participant information sheets were provided for each study to ensure informed consent. We balanced the need to achieve saturation with the duration and complexity of the project. As interviews progressed, consistent themes emerged among the participants in Part Two and recruitment ceased. We have broadly adhered to the guidelines of the Consolidated Criteria for Reporting Qualitative Research (COREQ) to report the key characteristics and methods of our study [23].

### Part One: Online Questionnaire

Respondents were recruited through professional organizations, social media advertisements, and snowball sampling methods. Individuals had to be registered health practitioners working in New Zealand mental health services or students enrolled in psychology or psychiatry training programs to participate in the questionnaire. In New Zealand, the law mandates that individuals working in specific health professions, including nursing, occupational therapy, psychology, and social work, must be registered with their respective regulatory bodies to practice legally [24]. The survey assessed participants’ training, experience, and perceived needs regarding digital mental health tools. Respondents could exit the questionnaire at any time, terminating their participation. Those who did not provide consent were thanked for their time, and the questionnaire was automatically closed.

The section of the survey about training needs contained 6 questions (see questions in Table 1). One question was conditional based on the answer to the previous question. There were 2 yes or no questions, 2 multiple-choice questions, and 2 five-point Likert scales, assessing if participants had received training, what kind of training it was, and what future training might be of interest.

**Table 1.** Part One survey questions.

Question	Answer option
Have you ever received training about digital mental health tools? (eg, an overview of available tools, a hands-on session or instruction on how to use them clinically)	Yes or no
What type of training was it? (Choose all that apply; conditional question, displayed only if answered “yes” to having received training)	Curriculum-part of undergraduate or postgraduate training On the job—onsite workshop or training session Offsite-conferences, seminar or webinar
Have you ever sought out to learn about digital mental health tools on your own? (eg, textbook, journal article, online resources or similar)	Yes or no
Do you think training is necessary to get the most from digital mental health tools?	5-point Likert—“Definitely not” to “Definitely yes”
How interested would you be in receiving training in digital mental health tools?	5-point Likert—“Not at all” to “A great deal”
What type of training for digital mental health tools would you be most interested in? (Choose all that apply)	Interactive online tutorial Webinars or podcasts by experts Government guidelines or information from health authorities Interactive workshops in person Informational booklets or reading materials, conferences including information on digital mental health tools Lectures given by experts Other

### Part Two: Follow-Up Interviews With Survey Respondents

At the conclusion of the online survey, participants were invited to express interest in a follow-up interview. Those who opted in were contacted by a researcher (CR), provided with written information about the study, and offered an opportunity to schedule an interview at a convenient time. Written informed consent was obtained via Google Forms prior to the interview. Participants were given the opportunity to ask questions about the study and withdraw at any time. All those who provided consent completed their interview. One-on-one interviews were conducted online using Zoom and lasted approximately 30-40 minutes. A semistructured interview guide (see questions in Textbox 1) was used to ensure consistency across interviews while allowing for flexibility to explore relevant topics in greater depth. Interviews were audio-recorded, transcribed verbatim, and anonymized for analysis. Participants were not given the opportunity to review their recordings or transcripts nor to provide feedback on the findings.

Textbox 1. **Questions for clinicians interviewed in Part Two.**

Have you received training in digital mental health tools?

**For those who have had training**

What kind of training have you had in digital mental health tools?

What was most or least helpful from this training?

Would you like more training? What kind? How can we improve existing training do you think?

**For those who have not had training**

Why do you think you have not received training in digital mental health tools? (Not keen, not available, no time, no funding, etc)

Would you like to get some training in the future? What kind?

What would encourage you to receive training in digital mental health tools?

### Part Three: In-Depth Interviews With Educators

Program coordinators from psychology and psychiatry training programs across New Zealand’s 7 universities were identified and contacted directly via email. In this study, educators were defined as individuals responsible for curriculum development and revision and those engaged in teaching or teaching coordination within their respective psychology or psychiatry courses. Recruitment was straightforward due to the limited number of relevant programs nationwide. We contacted 10 programs from the clinical, health, and counseling scopes, and 4 agreed to take part. Of the 2 psychiatry programs, 1 agreed to take part. Coordinators who expressed interest in participating were contacted by CR to arrange a short (less than 30 min) interview. The same consent, recording, and transcription procedures used in Part Two were applied. Interviews explored the extent to which digital mental health tools were incorporated into existing curricula, as well as perceived barriers and opportunities for further integration (see Textbox 2).

Textbox 2. **Questions for program coordinators in Part Three.**

Can you tell me about the training program you are involved in and your role within it?

Does your curriculum currently include any teaching on digital mental health tools?

What has been the student response to digital mental health tools—have they shown interest or asked about them?

How do you see the role of digital mental health tools evolving in New Zealand?

**For curricula that include digital tools**

What prompted you to include digital mental health tools in your curriculum?

What does the teaching on digital mental health tools look like (eg, guest classes, hands-on workshops, webinars)?

Have you prioritized any specific tools, and did this require dropping other content?

**For curricula without digital tools**

Do you intend to incorporate digital mental health tools into your curriculum?

What are the biggest barriers to adding digital mental health tools to the curriculum?

What would encourage or enable you to introduce digital mental health tools?

### Data Analysis

Data from the survey (Part One) were analyzed using descriptive and inferential statistics to identify trends and patterns in participants’ responses. Qualitative data from Parts Two and Three were transcribed using the Descript software (v32.1.0), with a researcher (CR) verifying each transcript for accuracy. Reflexive thematic analysis, guided by the 6-phase process [25], was conducted using NVivo software (release 1.61; Lumivero). The analysis began with familiarization through repeated readings and initial note-taking. Key features relevant to the research questions were then coded, and these codes were grouped into preliminary themes with the themes derived from the data. Over time, the themes were refined and named to best reflect the data. Both authors engaged in discussions throughout the process to finalize major themes. Given the interpretive nature of the analysis, no cross-coding was performed, prioritizing reflexivity over intercoder reliability [26]. This approach allowed for a deeper exploration of participants’ experiences. Verbatim quotes are included to highlight key findings, with participant qualifications provided for context.

## Results

### Part One (Online Questionnaire)

A total of 144 people started the survey, and 118 participants completed it. The demographic and professional characteristics of the participants are summarized in Table 2. Most (n=91, 77%) participants identified as female, with a significant proportion (n=65, 55%) aged between 31 and 50 years. Most (n=103, 87%) respondents reported working clinically across various settings, including public health services (n=60) and private practice (n=31). Participants represented a range of specialties, with clinical psychology (n=33, 28%) and psychiatry (n=22, 19%) being the most common.

For a detailed breakdown of participant demographics, including ethnicity, location, and professional background, readers are referred to the full description of the sample published here [21].

**Table 2.** Demographic and professional characteristics of Part One (survey) participants^a^.

Characteristic	Participants, n (%)
Gender	
Man	20 (17)
Woman	91 (77)
Gender diverse or prefer not to say	7 (6)
Total	118 (100)
Age (y)	
20‐40	52 (44)
41‐60	59 (50)
≥61 or prefer not to say	7 (6)
Total	118 (100)
Ethnicity	
Asian	11 (9)
Māori	9 (8)
NZ European or Pākehā	80 (68)
Pacific Islands or other	17 (15)
Total	117 (100)
Employment or training status	
Working clinically (full or part-time)	103 (87)
In training (full or part-time)	16 (13)
Academia or other	7 (6)
Total	126 (106)
Length of clinical practice (y)	
10‐19	25 (25)
≥20	33 (33)
Total	102 (102)
Area of work (Population)	
Adults	84 (82)
Infants, children, and adolescents	50 (49)
Older adults	34 (33)
Total	168 (164)
Specialty	
Clinical psychology	33 (28)
Psychiatry	22 (19)
Counseling	17 (14)
Nursing	13 (11)
Occupational therapy	12 (10)
Social work	12 (10)
Other	21 (17)
Total	130 (109)

a Percentages may not total 100% due to rounding and multiple responses. Not all questions were compulsory to answer.

### Training Needs and Preferences

Of the 118 clinicians who completed the survey, 75% (n=88) reported having no formal training in digital mental health tools. Despite this, 69% (n=81) had independently sought to learn about them. Among the 30 clinicians who had received training, 18 participated in offsite training (eg, conferences, seminars, or webinars), 17 underwent on-the-job training (eg, onsite workshops), and 5 received training during their undergraduate or postgraduate education. As shown in Table 3, most clinicians recognized the necessity of training to use digital mental health tools effectively and expressed significant interest in further education. Preferred training formats included interactive online tutorials, expert-led webinars, and guidelines from health authorities.

**Table 3.** Summary of clinician perspectives on training in digital mental health tools.

Question and response option	Participants, n (%)
Is training necessary for digital tools?	
Definitely not	1 (1)
Probably not	18 (15)
Unsure	7 (6)
Probably yes	65 (65)
Definitely yes	27 (23)
Total	118 (100)
Interest in receiving training	
Not at all	9 (8)
A little	11 (9)
Moderately	37 (31)
A lot	28 (24)
A great deal	33 (28)
Total	118 (100)
Preferred training formats	
Interactive online tutorial	86 (73)
Webinars or podcasts by experts	77 (65)
Government guidelines or health authority information	60 (51)
Interactive workshops in person	48 (41)
Informational booklets or reading materials	45 (38)
Conferences including digital tools info	37 (31)
Lectures given by experts	30 (25)
Conferences specifically on digital tools	28 (24)
Other	10 (8)

### Part Two (Follow-Up Interviews With Clinicians)

A total of 17 participants were involved in Part Two. Their demographic and professional characteristics are summarized in Table 4. For a detailed breakdown of participant demographics, readers are referred to the full description of the sample published here [22].

**Table 4.** Part Two clinician interviewee demographics^a^.

Characteristic	Participants, n (%)
Gender	
Man	2 (12)
Woman	15 (88)
Total	17 (100)
Age (y)	
21‐40	7 (41)
41‐60	8 (47)
≥61	2 (12)
Total	17 (100)
Ethnicity	
Asian	3 (18)
Māori	3 (18)
NZ European	9 (53)
Other (Irish and Romanian)	2 (12)
Total	17 (101)
Qualifications	
Psychiatrist	6 (35)
Clinical psychologist	3 (18)
Mental health nurse	3 (18)
Social worker and counselor	2 (12)
Social worker	1 (6)
Counselor	1 (6)
Occupational therapist	1 (6)
Total	17^b^ (101)
Use of digital tools	
Had used digital tools	13 (77)
Had not used digital tools	4 (24)
Total	17 (101)
Investigation of digital tools	
Had investigated digital tools	15 (88)
Had not investigated digital tools	2 (12)
Total	17 (100)

a Percentages in the table do not total 100% due to rounding only. All participants answered these questions.

b Two participants held both social work and counseling qualifications.

### Part Two Training Themes Identified

**Figure 1.**
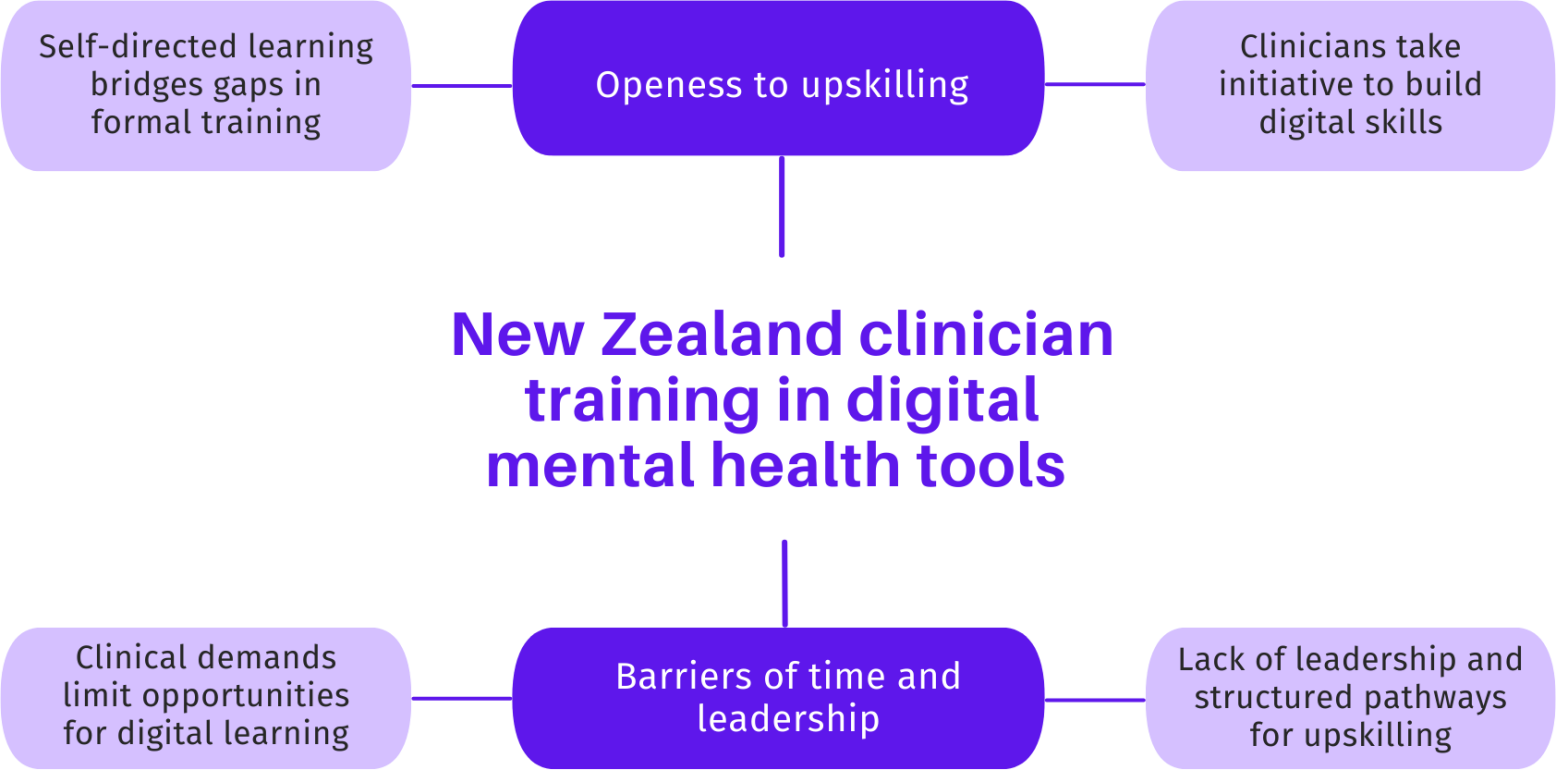
Part Two (clinician interview) themes and subthemes.

We have previously presented the findings from Part Two, which relate to knowledge and attitudes and contain different themes [22]. Two main themes regarding training needs and preferences were identified, each with 2 subthemes, as shown in Figure 1.

### Theme One: Openness to Upskilling

#### Overview

This theme reflects clinicians’ willingness to adopt digital mental health tools, even in the absence of formal training. Many have integrated these tools through self-directed learning, often prompted by client needs or professional curiosity. Despite this, clinicians expressed strong interest in structured training to ensure safe and effective use. They highlighted the need for practical, accessible sessions covering tool efficacy, risk management, and integration into practice, reflecting a proactive yet cautious approach to digital innovation.

#### Self-Directed Learning Bridges Gaps in Formal Training

Few interviewed clinicians had received formal training in digital mental health tools; however, most had experience using them with clients. When reflecting on the lack of training opportunities, clinicians noted that digital health is “a new evolving field” and has “probably not quite filtered down into the training curriculum.” Others highlighted that the health system is not yet prepared to prioritize digital health training over more immediate clinical needs.

*The system is very dated and stuck in its ways and struggling with adjusting and adapting to modern trends. Also, because sometimes people wait for certain evidence to come along before there is a change made, so instead, there’s no one really pushing trials or encouragement of support.* [Psychiatrist]

Clinicians expressed strong interest in receiving formal training if it became available, particularly to address concerns about the efficacy and safety of digital tools. They felt that this was not something they could—or wanted to—pursue independently, preferring structured training to ensure consistency and reliability. Despite this hesitance, many clinicians had already begun using digital tools to assist clients and provide additional resources. Formal training was seen as essential to increase confidence and improve the responsible integration of these tools into practice.

*Training would be great. Because I think that if we’re going to use this more often, which I’m very keen to do, that we have to be very aware, we have to find that balance between the relaxedness of using tools and the risk-benefit analysis, really making sure that you’re not going to cause any harm*. [Psychiatrist]

Clinicians were particularly interested in training that covered the background, efficacy, and safe use of digital tools, as well as guidance on integrating them into clinical settings—most preferred short, practical formats such as webinars or interactive online workshops.

*If we are all going to start using e-tools, there probably does need to be a type of process to be trained in them. Covering the basics like confidentiality, privacy, how to use them, who would be appropriate and when to stop using them. Someone who knows the work really well and the New Zealand context and what’s out there who works with clients and client feedback would be good to have*. [Clinical psychologist]

There was also interest in specific recommendations regarding which digital tools were appropriate for different diagnoses, situations, and populations. Clinicians wanted to be able to implement suitable tools in their practice immediately.

*I think the first important thing is to see what’s out there, and the next thing would be picking up a couple of the most popular tools and doing a brief run-through. Showing firstly what they do and how clients can use them at home and then sharing examples or ways people have thought of how you can integrate them into counselling or therapy sessions*. [Counselor]

#### Clinicians Take Initiative to Build Digital Skills

Clinicians became aware of digital mental health tools primarily through word of mouth from colleagues, professional networks, or clients who introduced them during sessions.

*A lot of the recommendations for apps come through clients in the first place because I guess they’re desperately seeking something. And then we usually download it onto work phones and check it out*. [Clinical psychologist]

After hearing about these tools, clinicians often took the initiative to explore their availability and suitability for clients. This process of investigation and adoption was largely self-directed, relying on personal judgment, clinical training, and occasional review of current research.

*When it comes to the use of things, most of it is self-learned or by trial and error or by recommendation from somebody else.* [Psychiatrist]

When clinicians identified digital tools they believed could benefit clients, they trialed them informally, seeking feedback on their effectiveness and ease of use.

*Using them myself, having a look, thinking about how it would apply to me and then asking clients too, how was that? or what didn’t you like? What did you like when you used it?* [Clinical psychologist]

Successful tools that resonated with clients were often shared within professional circles, contributing to the organic spread of resources across agencies and networks.

*I can share with other agencies and say, Hey, you could use this. Do you know about these apps? Do you know about this website? Because this might be helpful for your clients and everybody falls on it.* [Social worker and counselor]

### Theme Two: Barriers of Time and Leadership

#### Overview

This theme highlights the challenges clinicians face in adopting digital mental health tools due to limited time and lack of clear leadership. Despite their interest, heavy workloads and competing responsibilities prevent clinicians from evaluating tools independently. Without endorsement from health authorities, many are hesitant to integrate digital tools, particularly in public health settings where approval is required. The absence of leadership and institutional support shifts responsibility to individual clinicians, which many find overwhelming and impractical.

#### Clinical Demands Limit Opportunities for Digital Learning

Clinicians juggle numerous responsibilities and often face heavy workloads, leaving little capacity to independently evaluate the growing number of digital mental health tools for safety, efficacy, and ethical considerations. The sheer volume of available tools, coupled with limited time, makes it impractical for clinicians to vet each option thoroughly. Despite recognizing the potential benefits of digital interventions, many clinicians feel unequipped to take on the added responsibility of monitoring client use and mitigating potential risks without institutional support.

*I think having time to, like go through every single website or app, it’s just not feasible.* [Psychiatrist]

For those working within the public health system, the lack of formal approval processes further restricts the ability to integrate digital tools into clinical workflows. Clinicians cannot independently adopt or finance tools without institutional backing, adding another layer of complexity. In private practice, clinicians similarly seek reassurance that tools have been rigorously evaluated and deemed safe and effective by trusted experts. This need for validation reflects a broader desire for consistency and reliability across the sector, reducing the risk of harm and ensuring clients receive high-quality, evidence-based care.

*I work in a public service, so obviously, if I’m going to use something, it needs to be agreed upon by the DHB. That’s a big barrier. I can’t just bring up tools that I’ve found on the internet and paid for. The DHB has to pay for it.* [Psychiatrist]

#### Lack of Leadership and Structured Pathways for Upskilling

None of the clinicians interviewed had received formal guidance or structured training on digital mental health tools from their clinical leadership. While some were aware of growing interest from the Ministry of Health, this awareness had not translated into concrete recommendations or frameworks for implementation. Clinicians expressed a pressing need for explicit endorsement and clear guidelines from health authorities to ensure that the use of digital tools aligns with best practices and ethical standards. Without these endorsements, uncertainty persists, creating hesitation around adopting tools that could otherwise enhance care.

*There’s no library to easy access to be like, oh yeah, look, this is something the Ministry of Health supports. For example, in the UK, there’s the NHS digitals, and people have access to a government-approved list of resources where here there’s nothing like that.* [Psychiatrist]

There is a clear need for health organizations to lead the way by assessing, endorsing, and curating a selection of trusted digital tools for clinicians. This would remove the burden of evaluation from individual practitioners and ensure that recommended tools meet established clinical and ethical standards. Without this top-down guidance, clinicians are left to navigate digital health on their own, heightening the risk of inconsistent practices and potential harm to clients.

*Something we can use like a CBT course digitally delivered, would be really useful, but ultimately, there’s nothing that’s endorsed or supported by the DHB, which for me feels like the barrier. It’s quite overwhelming to expect the individual clinician to evaluate and then take on board that responsibility when there are some ethical considerations.* [Psychiatrist]

### Part Three: Interviews With Educators

A total of 4 participants were involved in Part Three, and their demographic and professional characteristics are summarized in Table 5.

**Table 5.** Part Three educator interviewee demographics^a^.

Characteristic	Participants, n (%)
Gender	
Man	1 (25)
Woman	3 (75)
Total	4 (100)
Age (y)	
21‐40	1 (25)
41‐60	3 (75)
Total	4 (100)
Specialty	
Psychology	3 (75)
Psychiatry	1 (25)
Total	4 (100)

a As New Zealand has few training programs, we are unable to report ethnicity or further details of specialty as anonymity could be compromised.

### Part Two Training Themes Identified

**Figure 2.**
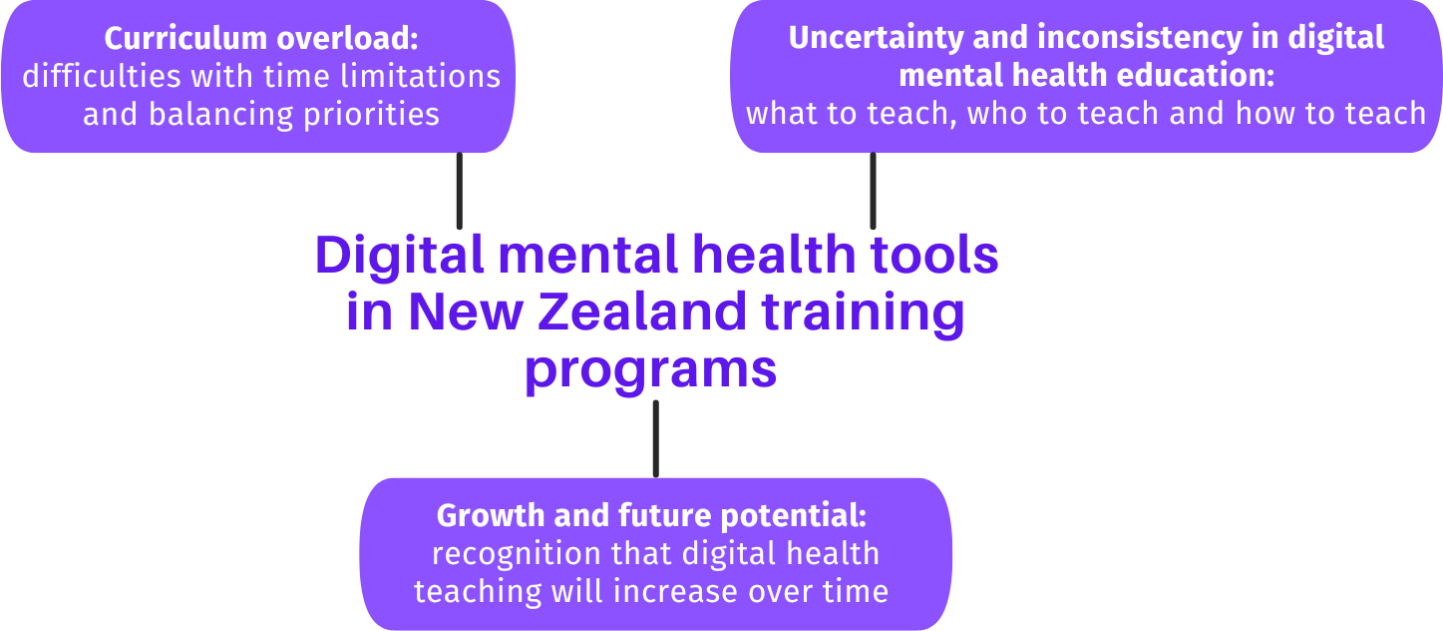
Part Three educator themes.

A total of 3 main themes regarding the inclusion of digital health into training programs were identified (Figure 2).

### Theme One: Curriculum Overload

This theme relates to significant challenges in integrating new content, such as digital mental health tools, into existing psychology and psychiatry training programs. With limited time and space, curricula are already densely packed with essential topics that provide the foundation for clinical practice. The pressure to remain clinically relevant further complicates the introduction of digital health, as educators prioritize content that students can immediately apply in real-world settings. While digital health is recognized as important for the future, its inclusion should not come at the expense of other critical areas. Overall, while digital health is acknowledged as a growing and necessary component of mental health care, educators expressed the need for strategic planning to ensure it is integrated in a way that complements, rather than competes with, existing critical content.

To start with, the educators we spoke with noted the difficulty of fitting new material into already crowded curricula. As clinical practice evolves and new knowledge emerges, there is continuous pressure to update and adapt courses. However, participants highlighted that adding content, such as digital health, often requires removing or reducing other subjects—a complex and challenging process.

*I think we’d have to remove things, have to stop some stuff and start other stuff, and that transitional process is always very difficult. There’s just been more and more and more coming in, more demand to do more stuff.* [Educator in a psychology training program]

Another point raised by the educators is the importance of ensuring that what is taught remains clinically relevant. Digital mental health is an emerging area but is not yet fully embedded in everyday clinical practice. Educators expressed concern that if students cannot immediately apply digital health skills after graduation, the value of teaching it could be diminished. For digital health to be integrated, there must be confidence that the tools and knowledge will be directly usable in practice.

*I think whatever we teach, the registrars need to be able to use the new learning in their everyday practice because otherwise, the teaching of new topics or new skills, so how often can we apply the teaching in our clinical practice? So it’s about how to link that with the clinical services, the education so the clinicians can apply it in their everyday work because otherwise, people will just lose interest, won’t they with idle skills.* [Educator in a psychiatry training program]

A key concern is ensuring that training remains broad enough to meet the needs of various subspecialties in mental health. The volume of content that must be covered in training is substantial, ranging from neuroscience and trauma work to child and family psychology. Educators emphasized that the scope of training is so extensive that incorporating digital health content feels difficult to justify within the constraints of current programs.

*Even in our training program, we have so much to cover. We have to do neuroscience, we have to do child psychology, have to do child and family work, have to do adult work, trauma work, and so you literally struggle to fit everything that we need to cover for general psychologists into our program.* [Educator in a Psychology Training Program]

### Theme Two: Uncertainty and Inconsistency in Digital Mental Health Education

The educators highlighted significant variability in how digital mental health content is integrated into training programs. Most programs incorporate this content into existing courses rather than offering dedicated modules, often limiting coverage to 1 or 2 sessions focusing on telehealth and ethical considerations. This reflects the growing reliance on video conferencing during the COVID-19 pandemic. Only 1 program offered a semester-long course providing comprehensive exposure to digital mental health.

*It’s not extensive. It’ll be more kind of woven into the program where it’s relevant, and maybe one or two sessions specifically focused on that. […] The big focus is on video conferencing, like Zoom.* [Educator in a psychology training program]

The inclusion of digital mental health content is typically driven by educators who recognize its growing importance rather than by direct student demand. Some students enter training with prior experience in digital interventions, reducing perceived teaching needs. However, feedback on the value of this content is inconsistent, leaving educators unsure about its emphasis in curricula.

*The technology, I haven’t heard anyone yet expressing an interest. […] They’ve all kind of done that online engagement around distress and counselling and so on.* [Educator in a psychology training program]

Another challenge is the rapid evolution of digital mental health, with new tools emerging regularly. Educators reported difficulty keeping teaching materials up-to-date and ensuring relevance amid the overwhelming variety of available tools.

*There are so many varieties we are talking about, and people get confused with technology. […] What digital mental health tools should we use?* [Educator in a psychiatry training program]

The educators also noted hesitancy to endorse specific tools due to commercial ties, which may conflict with the academic emphasis on neutrality.

*I think there’s a tension […] not to be seen to promote certain products. […] We have to be careful in terms of not recommending particular programs or websites, if they’ve got some kind of commercial aspects.* [Educator in a psychology training program]

A lack of expertise among teaching staff further complicates efforts to expand digital mental health education. Many educators noted that the responsibility for this content often falls on those already stretched thin, with some clinicians hesitant to engage with digital tools.

*Then, of course, you need people to teach it because those have to be people who are familiar with this medium, too. […] A lot of psychologists teaching are a little older, been around for a while feeling no, this is unfamiliar territory for us.* [Educator in a psychology training program]

### Theme Three: Growth and Future Potential

Educators universally acknowledged that digital mental health is a rapidly expanding field with increasing relevance to clinical practice. As digital interventions become more integrated into mental health services, educators expect digital health to take on a greater role in training curricula. They anticipate that, over time, there will be more dedicated teaching to ensure students are adequately prepared for the realities of clinical work. Without this knowledge, graduates risk being underprepared for the evolving demands of day-to-day practice.

*I think in the end, it’s about coming to that point where you realise this is a priority, and so we have to make space for it. It has to be there. And I think that’s one good thing. The only good thing is that COVID might have forced us into thinking about that.* [Educator in a psychology training program]

Educators highlighted that the continued growth of digital mental health is inevitable and warned that failing to incorporate it into training could leave students at a disadvantage. They emphasized the need to familiarize students with available tools and interventions to ensure they are equipped to navigate the digital landscape upon entering the workforce.

*Well, I think it’s a space that’s just going to keep growing, and I think that if we don’t teach our students about this, they’re actually going to be quite ill-equipped when they enter the workforce. They don’t know what’s out there.* [Educator in a psychology training program]

## Discussion

### Principal Findings

The findings from the survey (Part One) revealed that three-quarters of clinicians lacked formal training in digital mental health tools, though the majority (69%) had independently sought to learn about them. Reported training was often informal, occurring through offsite conferences, webinars, or self-directed efforts, with minimal integration into academic programs. Clinicians broadly agreed that training is essential to effectively utilize digital mental health tools, and interest in such training was notably high. Preferred formats included interactive online tutorials, expert-led webinars or podcasts, and official guidelines from health authorities.

Despite strong interest in upskilling, follow-up interviews (Part Two) revealed significant barriers to engaging in digital mental health training. Clinicians frequently cited heavy workloads and competing responsibilities as key obstacles, limiting their capacity to explore or evaluate digital tools. A lack of leadership and institutional support further compounded hesitancy around adopting digital interventions, particularly in public health settings where clinicians were reluctant to implement tools without approval from governing bodies. The absence of official guidance left clinicians feeling burdened by the need for self-directed learning, leading to inconsistencies in the adoption of digital health tools.

The educators (Part Three) recognized the importance of integrating digital health into student training. However, they highlighted challenges such as overloaded curricula and the need for educators to stay current with digital health innovations or rely on external expertise. They also highlighted the importance of continuous learning to keep pace with technological advancements, underscoring the need for flexible, responsive training models rather than one-off, static courses. There was optimism that as tools become more widely accepted and used, formalized training will follow. This perspective suggests that while digital health training faces immediate challenges, the long-term trajectory points toward increasing adoption and curriculum inclusion.

Given the constraints of packed curricula and heavy clinical workloads, our findings support the implementation of concise, on-demand training (eg, prerecorded sessions with supporting materials) that is directly applicable to clinical practice. Such training should be readily accessible to both students and fully qualified clinicians across the health system. Preferred formats, such as interactive online tutorials and webinars or podcasts, underscore the importance of flexible, accessible, on-the-job learning. The increased adoption of webinars and distance learning during the global pandemic highlights an opportunity to meet this demand, as clinicians have shown a growing interest in engaging with digital education. Advances in online learning technology further support the efficient delivery of these training initiatives [11,27].

Our findings align with existing literature, highlighting persistent gaps in clinician training for digital mental health tools. Two New Zealand studies by Van Kessel [28] and Wilson [29] identified limited knowledge as a key barrier to adoption in New Zealand, with significant clinician interest in training. The first study by Van Kessel [28] reported that 75% of the clinical psychologists were interested in training that and 78% were willing to use digital tools if provided with training. Similarly, we found that 83% (n=92) of the respondents believed training was necessary, and over half (n=63) expressed strong interest, underscoring a pervasive, cross-disciplinary need for training. The findings from Van Kessel [28] combined with our study highlight the importance of targeted, practical training formats, such as online tutorials, expert-led webinars, and guidelines from health authorities. Our results extend these insights by emphasizing the need for flexible, time-efficient training that accommodates the demands of students and practicing clinicians. However, despite growing recognition of digital health’s importance, systemic progress has been limited, hindered by crowded curricula, lack of institutional leadership, and educator expertise. This reinforces the need for coordinated efforts to address structural barriers and bridge the training gap.

Wilson [29] also emphasized the critical role of health professionals in promoting digital interventions, noting that clinician recommendations significantly increase public uptake. Similarly, our findings suggest that training can positively influence clinician attitudes and confidence, driving higher adoption rates. Survey respondents and interview participants expressed enthusiasm for digital health tools but also conveyed feelings of uncertainty and a lack of support from employers and health authorities. This suggests that systemic leadership and investment are critical for fostering digital health adoption.

Our findings also resonate with international literature. Research in Germany [30] found that while clinicians lacked familiarity with eHealth tools, they were interested in training and recognized the potential benefits of digital interventions for prevention and self-help. Across the European context, similar themes were highlighted, where organizations recognized the potential of digital treatments to improve access and reduce costs but faced challenges such as integration difficulties and limited organizational knowledge [31]. These studies align with our findings. New Zealand clinicians have reported a strong desire for training and highlighted the absence of structural and leadership support, leaving them to navigate adoption individually.

In Australia, Sturk et al [32] examined clinician attitudes toward the Head to Health website, a government initiative providing digital mental health tools, and found that training played a key role in its adoption. While most clinicians were initially unaware of the resource, many became willing to recommend it to clients after receiving training. Consequently, participants called for broader promotion and better integration of digital tools into health care systems. Similarly, New Zealand clinicians in our study emphasized the importance of training and system-wide guidance to build confidence and increase the adoption of digital tools. These parallels suggest shared regional opportunities to foster digital mental health adoption through targeted training and systemic integration.

Significant variation in teaching digital competencies was revealed across German medical schools, highlighting the need for well-defined training to ensure clinical competence. Personal use of digital technology does not guarantee clinical proficiency, a sentiment echoed in our findings [33]. New Zealand clinicians strongly advocated for accessible, structured training, emphasizing the need for systemic leadership and investment to ensure clinicians are equipped to integrate digital tools into practice effectively [16,34].

Finally, a review focusing primarily on research in the United States further emphasized the importance of targeted digital literacy training for health workers, demonstrating its potential to advance digital health equity. This review highlighted that such training consistently delivers promising outcomes, whether broad or tool-specific [35]. Similarly, our findings reveal a strong demand among New Zealand clinicians for short, accessible training formats tailored to their clinical needs, reinforcing the global call for practical digital health education.

### Strengths and Limitations

This study’s primary strength lies in its mixed methods design, combining survey data with in-depth interviews to comprehensively explore digital mental health training needs and preferences among the New Zealand mental health workforce. Including multiple perspectives—clinicians, trainees, and educators—allowed for a nuanced understanding of both frontline experiences and systemic challenges within training programs.

Despite these strengths, the study has several limitations. The sample size for the follow-up interviews (n=17) and educator interviews (n=4) is small, limiting the generalizability of the findings. In particular, the small educator sample means the views captured may not represent the full diversity of perspectives across training institutions.

The sample of participants was skewed toward older and female participants, reflecting broader workforce demographics but potentially limiting insight into the perspectives of younger clinicians, who may have different experiences with technology. As generational shifts occur, younger clinicians will likely bring greater digital literacy and enthusiasm for digital tools, which may influence future training needs.

The clinicians who opted to participate in follow-up interviews may have been particularly vocal, concerned, or enthusiastic about digital mental health, potentially resulting in an overrepresentation of specific viewpoints. Additionally, they may have been primed by the survey questions, influencing the themes that emerged during the qualitative phase of the study [3,15,36].

### Conclusions

This study underscores the pressing need for enhanced training in digital mental health tools for clinicians and trainees in New Zealand. The findings reveal that while clinicians recognize the value of digital tools, most have not received formal training and instead rely on self-directed learning. This reflects not only the proactive nature of clinicians but also the lack of structured, accessible training pathways. Educators similarly acknowledged the importance of integrating digital health into curricula but face barriers due to already overloaded programs.

To address these challenges, the following key recommendations emerge:

- *Endorsement and leadership from health authorities*: Official endorsement of digital tools and training by governing health bodies is essential to build clinician confidence. Clear guidance and curated lists of approved tools would help ensure digital interventions align with best practices and ethical standards, reducing hesitation around their adoption.
- *Ongoing and flexible education*: Given the rapid evolution of digital health technologies, training must be continuous and adaptable. Regular updates, refresher courses, and emerging topics such as artificial intelligence−driven tools should be integrated into professional development. Training should be delivered in flexible formats, including interactive online modules, expert-led webinars, and self-paced learning materials that accommodate clinicians’ busy schedules.
- *Workplace-integrated learning*: Digital health training should be embedded in both formal education and on-the-job learning opportunities to ensure clinicians remain proficient throughout their careers. Short, practical sessions—such as lunchtime webinars or microlearning modules—can facilitate upskilling without significantly disrupting patient care. For those already in practice, structured workplace training is crucial to keep pace with advancements in digital health.

By addressing these areas, digital mental health tools can become more embedded in clinical workflows, improving patient outcomes and streamlining service delivery. These recommendations reflect not only clinician preferences but also the broader systemic need to support digital innovation in mental health care through structured, responsive, and practical training initiatives.

## Abbreviations

COREQ: Consolidated Criteria for Reporting Qualitative Research
